# Using HVS Dual-Pathway and Contrast Sensitivity to Blindly Assess Image Quality

**DOI:** 10.3390/s23104974

**Published:** 2023-05-22

**Authors:** Fan Chen, Hong Fu, Hengyong Yu, Ying Chu

**Affiliations:** 1 Department of Artificial Intelligence, Shenzhen University, Shenzhen 518060, China; 2070276166@email.szu.edu.cn; 2 Department of Mathematics and Information Technology, The Education University of Hong Kong, Hong Kong, China; hfu@eduhk.hk; 3 Department of Electrical and Computer Engineering, University of Massachusetts Lowell, Lowell, MA 01854, USA; hengyong_yu@uml.edu

**Keywords:** no-reference image quality assessment, dual-stream networks, contrast sensitivity, ventral pathway, dorsal pathway

## Abstract

Blind image quality assessment (BIQA) aims to evaluate image quality in a way that closely matches human perception. To achieve this goal, the strengths of deep learning and the characteristics of the human visual system (HVS) can be combined. In this paper, inspired by the ventral pathway and the dorsal pathway of the HVS, a dual-pathway convolutional neural network is proposed for BIQA tasks. The proposed method consists of two pathways: the “what” pathway, which mimics the ventral pathway of the HVS to extract the content features of distorted images, and the “where” pathway, which mimics the dorsal pathway of the HVS to extract the global shape features of distorted images. Then, the features from the two pathways are fused and mapped to an image quality score. Additionally, gradient images weighted by contrast sensitivity are used as the input to the “where” pathway, allowing it to extract global shape features that are more sensitive to human perception. Moreover, a dual-pathway multi-scale feature fusion module is designed to fuse the multi-scale features of the two pathways, enabling the model to capture both global features and local details, thus improving the overall performance of the model. Experiments conducted on six databases show that the proposed method achieves state-of-the-art performance.

## 1. Introduction

With the rapid development of digital multimedia technology and the popularity of various photography devices, image information has become an important source of human visual information. However, in the process of going from obtaining digital images to arriving at the human visual system, there is an inevitable degradation in image quality. Therefore, it is meaningful to research image quality assessment (IQA) methods that are highly consistent with human visual perception [1].

According to the degree of participation of the original image information, objective IQA methods can be classified into the following categories: full-reference IQA, reduced-reference IQA, and no-reference IQA [2]. No-reference IQA is also called blind IQA (BIQA). Because BIQA methods do not require the use of reference image information and are more closely related to actual application scenarios, they have become a focus of research in recent years [3].

Traditional BIQA methods (e.g., NIQE [4], BRISQUE [5], DIIVINE [6], and BIQI [7]) typically extract low-level features from images and then use regression models to map them to image quality scores. The extracted features are often manually designed and are often inadequate to fully characterize the quality of images. With the development of deep learning, many deep-learning-based BIQA methods (e.g., IQA-CNN [8], DIQaM-NR [9], DIQA [10], HyperIQA [11], DB-CNN [12], and TS-CNN [13]) have been proposed. With their powerful learning abilities, these methods can extract the high-level features of distorted images, and their performance is greatly improved compared to the traditional methods. Although most existing deep-learning-based IQA methods enhance the feature-extraction ability by proposing new network structures to improve the model’s performance, they overlook the important influence of HVS characteristics and the guiding role they may play.

The goal of BIQA is to judge the degree of image distortion with high consistency to human visual perception. It is natural to combine the characteristics of the human visual system (HVS) with powerful deep learning methods. Moreover, based on HVS characteristics, research on BIQA can provide new research perspectives for the study of IQA. This can help to develop evaluation metrics that are more in line with HVS characteristics and provide useful references for understanding how the HVS perceives image degradation mechanisms, making it a valuable scientific problem.

The HVS has many characteristics, such as the dual-pathway feature [14,15], in which visual information is transmitted through the ventral pathway and dorsal pathway in the visual cortex. The former is involved in image-content recognition and long-term memory and is also known as the “what” pathway. The latter is involved in processing spatial-location information of objects and is also known as the “where” pathway. Inspired by the ventral and dorsal pathways of the HVS, Karen and Andrew [16] proposed a dual-stream convolutional neural network (CNN) structure and successfully applied it to the field of video action recognition. They used a spatial stream to take video frames as input to learn scene information and a temporal stream to take optical flow images as input to learn object motion information. Optical flow images explicitly describe the motion between video frames, eliminating the need for CNNs to implicitly predict object motion information, simplifying the learning process, and significantly improving the model accuracy. The contrast sensitivity characteristic of the HVS reflects the different sensitivity of the human eye to different spatial frequencies [17]. This characteristic is similar to the widely used spatial attention mechanism [18] and image saliency [19]. Campbell et al. [20] proposed a contrast sensitivity function to explicitly calculate the sensitivity of the HVS to different spatial frequencies. Some traditional IQA methods [21,22] use the contrast sensitivity function to weight the extracted features to achieve better results. In addition, when perceiving images, the HVS simultaneously pays attention to both global and local features [23]. This characteristic is particularly important for IQA because the degree of distortion of authentically distorted images is often not uniformly distributed [24]. Some IQA methods [25,26] are designed for extracting multi-scale features based on this characteristic, and the results show that using multi-scale features can effectively improve the algorithm’s performance. The aforementioned HVS characteristics have been directly or indirectly applied to computer-vision-related tasks and have been experimentally proven to be effective.

The main contribution of this article is to propose a new model based on dual-pathway and contrast sensitivity (DPCS) for BIQA. The HVS’s dual-pathway characteristic is used to guide the construction of a dual-pathway BIQA deep learning model, which can simultaneously learn the content and spatial location information of distorted images. The multi-scale and contrast sensitivity characteristics of the HVS are also introduced to enable the model to extract distortion features that are highly consistent with human perception. Specifically, our contributions are as follows:

- First, inspired by the ventral and dorsal pathways of the HVS, a dual-stream convolutional neural network is proposed, with the two streams named the “what” pathway and the “where” pathway, respectively. The “what” pathway extracts the content features of distorted images, while the “where” pathway extracts the global shape features. The features of the two streams are fused and mapped into an image quality score.
- Second, by weighting the gradient image of the contrast sensitivity as the input of the “where” pathway, the global shape features that are sensitive to the human eye can be extracted.
- Third, a dual-stream multi-scale feature fusion module is designed to fuse the multi-scale features of the two pathways, enabling the model to focus on both global and local features of distorted images.

The rest of this paper is organized as follows. Section 2 introduces related works for BIQA and analyzes their limitations. Section 3 provides a detailed description of the proposed HVS-based dual-stream model, image-preprocessing method, and dual-stream multi-scale feature fusion module. Section 4 reports the experiment results. Section 5 discusses some related issues and concludes this paper.

## 2. Related Works

According to the method for feature extraction, BIQA methods can be generally divided into two categories: handcrafted feature-extraction methods and learning-based methods. Handcrafted feature-extraction methods typically extract the natural scene statistics (NSS) features of distorted images. Researchers have found that the NSS features vary with the degree of distortion. Therefore, NSS features can be mapped to image quality scores through regression models.

Early NSS methods extracted features in the transform domain of the image. For example, the BIQI method proposed by Moorthy and Bovik [7] performs a wavelet transform on the distorted image and fits the wavelet decomposition coefficients using the generalized Gaussian distribution (GGD). They first determine the type of distortion and then predict the quality score of the image based on the specific distortion type. Later, they extend the features of BIQI to obtain the DIIVINE [6], which more comprehensively describes scene statistics by considering the correlation of sub-bands, scales, and directions. The BLIINDS method proposed by Saad et al. [27] performs a discrete cosine transform (DCT) on distorted images to extract contrast and structural features based on DCT, which are then mapped to quality scores through a probabilistic prediction model. It is computationally expensive for all of these methods to extract features in the transform domain of the image. To avoid transforming the image, many researchers have proposed methods to directly extract NSS features in the spatial domain. The BRISQUE method proposed by Mittal et al. [5] extracts the local normalized luminance coefficients of distorted images in the spatial domain and quantifies the loss of “naturalness” of distorted images. This method has very low computational complexity. Based on the BRISQUE, Mittal et al. proposed NIQE [4], which uses multivariate Gaussian models (MVGs) to fit the NSS features of distorted and natural images and defines the distance between the two models as the quality of the distorted image. The handcrafted feature-extraction methods achieve a good performance on small databases (such as LIVE [28]), but the designed features can only extract low-level features of images, and their expressive power is limited. Therefore, their performance on large-scale synthetically distorted databases (such as TID2013 [29] and KADID-10k [30]) and authentically distorted databases (such as LIVE Challenge [31]) is relatively poor.

With the successful applications of deep learning methods to other visual tasks [32,33], more and more researchers have applied deep learning to BIQA. Kang et al. [8] first used CNNs for no-reference image quality assessment. To solve the problem of insufficient data, they segmented the distorted images into non-overlapping 32 × 32 patches and assigned each patch a quality score as its source image’s score. Bosse et al. [9] proposed DIQaM-NR and WaDIQaM-NR based on the VGG [32]. This method uses a deeper CNN and simultaneously predicts the quality scores and weights of image patches, and weighting summation is used to obtain the quality score of the image. Kim et al. [33] proposed BIECON. It uses the FR-IQA method to predict the quality scores of distorted image patches, utilizes these scores as intermediate results to train the model, and subsequently finely tunes the model using ground truth scores of images. Kim et al. [10] subsequently proposed DIQI. The framework is similar to BIECON but uses error maps as intermediate training targets to avoid overfitting. Su et al. [11] proposed HyperIQA for authentically distorted images. This method predicts the image quality score based on the perceived image content and also increases the multi-scale features so that the model can capture local distortions. Some researchers have introduced multitask learning into BIQA, which integrates multiple tasks into one model for training and promotes each other based on the correlation between tasks. Kang et al. [34] proposed IQA-CNN++, which integrates image quality assessment and image distortion type classification tasks and improves the model’s distortion type classification performance through multitask training. Ma et al. [35] proposed MEON, which simultaneously performs distortion-type classification and quality score prediction. Unlike other multitask models, the authors first pre-train the distortion-type classification sub-network and then perform joint training of the quality score prediction network. The experimental results show that this pre-training mechanism is effective. Sun et al. [36] proposed a Distortion Graph Representation (DGR) learning framework called GraphIQA. GraphIQA enables the distinction of distortion types by learning the contrast relationship between different DGRs and inferring the ranking distribution of samples from various levels within a DGR. Experimental results show that GraphIQA achieves state-of-the-art performance on both synthetic and authentic distortions. Zhu et al. [37] proposed a meta-learning-based NR-IQA method named MeataIQA. The method collects a diverse set of NR-IQA tasks for different distortions and employs meta-learning to capture prior knowledge. The quality prior-knowledge model is then fine-tuned for a target NR-IQA task, achieving superior performance compared to state-of-the-art methods. Wang and Ma [38] proposed an active learning method to improve the NR-IQA methods by leveraging group maximum differentiation (gMAD) examples. The method involves pre-training a DNN-based BIQA model, identifying weaknesses through gMAD comparisons, and fine-tuning the model using human-rated images. Li et al. [39] proposed a normalization-based loss function, called “Norm-in-Norm” for NR-IQA. The loss function utilizes the normalization of predicted and subjective quality scores and is defined based on the norm of the differences between these normalized values. Theoretical analysis and experimental results show that the embedded normalization enhances the stability and predictability of gradients, leading to faster convergence. Zhang et al. [40] conducted the first study on the perceptual robustness of NR-IQA models. The study identifies that conventional, knowledge-driven NR-IQA models and modern DNN-based methods lack inherent robustness against imperceptible perturbations. Furthermore, the counter-examples generated by one NR-IQA model do not efficiently transfer to falsify other models, highlighting valuable insights into the design flaws of individual models.

In recent years, continual learning has achieved significant success in the field of image classification, and some researchers have also applied it to IQA. Zhang et al. [41] formulated continual learning for NR-IQA to handle novel distortions. The method allows the model to learn from a stream of IQA datasets, preventing catastrophic forgetting and adapting to new data. Experimental results show the effectiveness of the proposed method compared to standard training techniques for BIQA. Liu et al. [42] proposed a lifelong IQA (LIQA) method to address the challenge of adapting to unseen distortion types by mitigating catastrophic forgetting and learning new knowledge without accessing previous training data. It utilizes the Split-and-Merge distillation strategy to train a single-head network for task-agnostic predictions. To enhance the model’s feature extraction ability, some researchers have proposed a dual-stream CNN structure. Zhang et al. [12] proposed a DB-CNN, which uses VGG-16, pre-trained on ImageNet [43], to extract authentic distortion features and uses CNN, pre-trained on Waterloo Exploration Database [44] and PASCAL VOC 2012 [45], to extract synthetic distortion features. Yan et al. [13] also proposed a dual-stream method. The two streams take the distorted image and its gradient image as input, respectively, so that the gradient stream focuses more on the details of the distorted image.

Although the aforementioned deep-learning-based BIQA methods have achieved good results, there is still room for further improvement. For example, the relevant characteristics of the HVS can be combined with deep learning to make the model consistent with the perceptual approach of the HVS. Inspired by the dual-pathway characteristics of the HVS, our work also adopts a dual-pathway structure. However, our two pathways extract the content features and location features of the distorted image, which are functionally consistent with the ventral and dorsal pathways of the HVS. In addition, our dual-pathway model adds contrast-sensitivity-weighted gradient images as an input. This provides different perspectives of the distorted image for the model and explicitly learns the contrast sensitivity characteristics of the HVS. The dual-pathway multi-scale feature fusion module designed in our work enables the model to focus on the global and local features of the image simultaneously. It is also highly consistent with the process of HVS perception.

In comparison to DB-CNN and TS-CNN, particularly TS-CNN, our method shares similarities in using gradient images as the input for one stream of the network. However, there are key differences between our proposed method and these two works. First, our method explicitly models both the ventral (“what” pathway) and dorsal (“where” pathway) streams of the human visual system, providing a more comprehensive representation of the human perception mechanism. Secondly, we introduce a contrast-sensitive weighting scheme for the gradient images in the “where” pathway, which enhances the sensitivity of the network to important contrast information in the input images. Thirdly, our dual-pathway multi-scale feature fusion module allows for the effective integration of features at different levels, enabling the network to capture both local and global image characteristics. These main differences contribute to the distinctiveness of our proposed method to DB-CNN and TS-CNN and enhance the ability of the proposed deep network to capture and evaluate image quality from the angle of human visual perception.

## 3. Proposed Method

**Figure 1:**
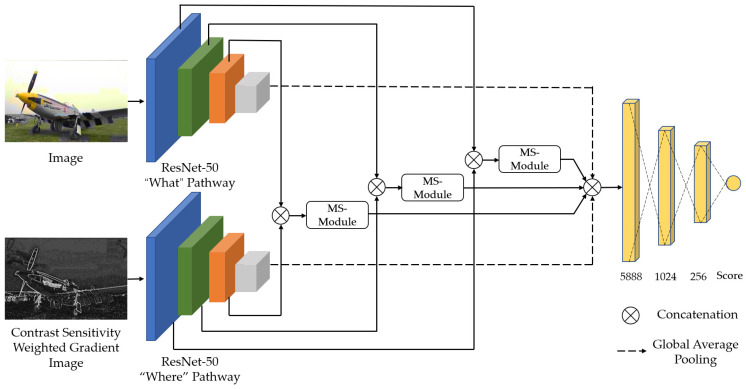
The architecture of the proposed method. MS-Module represents the multi-scale module.

Inspired by the ventral and dorsal pathways of the HVS, this paper proposes a dual-stream CNN structure for BIQA. The model architecture is shown in Figure 1, where the two pathways are referred to as the “what” pathway and the “where” pathway. Han and Sereno [46,47] proved that when modeling the ventral and dorsal pathways using CNNs, both pathways can use the same network structure. Therefore, here, both pathways have the same structure and use the ResNet-50 [48] as the backbone network, which is pre-trained on ImageNet. However, these two pathways receive as input distorted images and contrast-sensitivity-weighted gradient images, respectively, to achieve the function of the ventral and dorsal pathways. In addition, the model introduces a multi-scale feature fusion module that concatenates the multi-scale feature maps of the two streams and fuses them through the module. This allows the model to focus on the global features and local details of the image simultaneously.

### 3.1. “What” Pathway and “Where” Pathway

The “what” pathway takes a distorted image as the input and extracts content features through a pre-trained ResNet-50. The pre-trained ResNet-50 has demonstrated excellent performance in image classification tasks, proving its strong ability to understand image content. Because the content and structure of an image are closely related to its perceived quality, using ResNet-50 can better capture details and structural information in images, thus improving the accuracy of the model. To apply it to our method, the last average pooling layer and fully connected layer of the original ResNet-50 are removed, as shown in Table 1.

In line with the “what” pathway, the “where” pathway also uses the pre-trained ResNet-50 as a feature extractor. However, the “where” pathway takes a gradient image weighted by contrast sensitivity as the input. Gradient images provide rich structural and contour information, and the HVS is highly sensitive to such information [49]. Using gradient images allows the “where” pathway to extract object shape information from the distorted image, which is more consistent with the global shape perception of the dorsal pathway [50].

**Figure 2:**
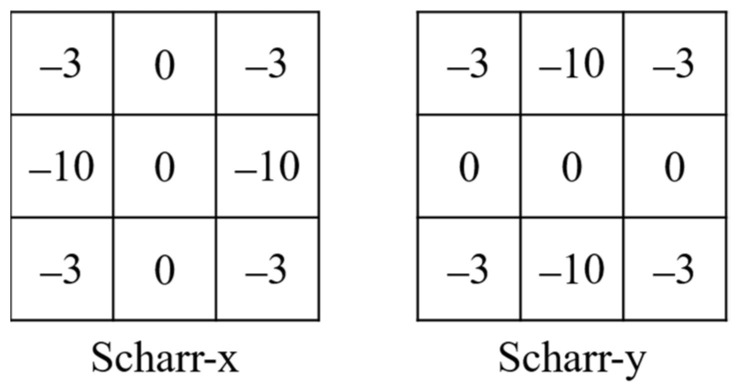
The structure of the Scharr operator.

The Scharr operator is a widely used edge-detection filter in computer vision and image processing. It is specifically designed to capture edges and gradients in images with high accuracy and low computational complexity. Compared to other popular image filters such as the Sobel and Roberts operators, the Scharr operator can offer superior performance in terms of edge detection and gradient estimation. Specifically, the Scharr operator utilizes a 3 × 3 kernel that approximates the derivative of an image using second-order differences. This kernel provides better isotropy properties, which means it can detect edges with equal sensitivity in all directions. This characteristic is particularly valuable for image quality assessment, as it allows for capturing edge information in distorted images accurately. Therefore, the Scharr operator [51] is chosen as the gradient operator, and its mask structure is shown in Figure 2.

The HVS has the characteristic of contrast sensitivity, meaning that the sensitivity of the human eye varies for different spatial frequencies. This characteristic is similar to the widely used spatial attention mechanism [18] and image saliency [19]. Campbell et al. [20] proposed a contrast sensitivity function to explicitly calculate the sensitivity of the HVS for different spatial frequencies:

(1) $$A\left(f\right)=2.6\left(0.192+0.114f\right)e^{-{\left(0.114f\right)}^{1.1}},$$

where f denotes the spatial frequency of a point. For the point $I\left(i,j\right)$, its spatial frequency can be calculated as:

(2) $$f=\sqrt{f_{x}^{2}+f_{y}^{2}},$$

(3) $$f_{x}=I\left(i,j\right)-I\left(i-1,j\right),$$

(4) $$f_{y}=I\left(i,j\right)-I\left(i,j-1\right).$$

The proposed method performs contrast-sensitivity weighting on gradient images to enhance the frequency information that is sensitive to the HVS, thereby making the model highly consistent with the HVS perception. Specifically, a contrast-sensitivity function is used to calculate the contrast sensitivity of each pixel in the distorted image. This yields a contrast-sensitivity image, which is then combined with the gradient image to obtain the contrast-sensitivity-weighted gradient image:

(5) $$I_{CWG}=\alpha I_{C}+\beta I_{G}+\gamma ,$$

where $I_{C}$ denotes the contrast sensitivity image, $I_{G}$ denotes the gradient image, and α, β, and γ are constants. We set α = β = 0.5 and γ = 0.

**Figure 3:**
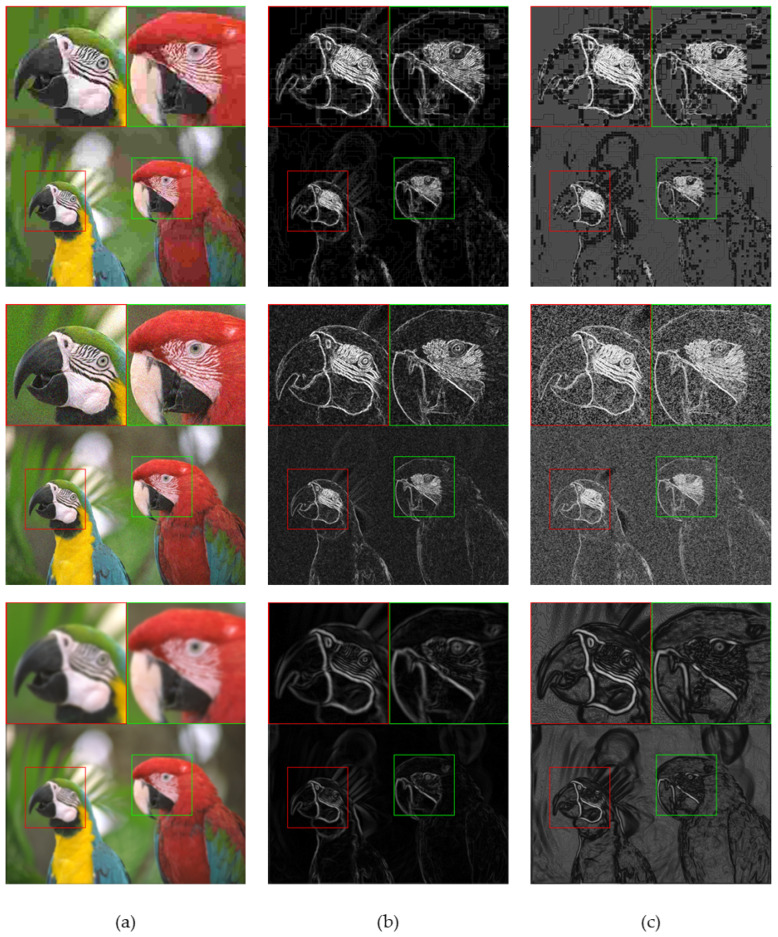
Examples of gradient images and the corresponding contrast-sensitive weighted gradient images for different distortion types. (**a**) are the distorted images with JPEG compression distortion, white Gaussian noise (WN) distortion, and Gaussian blur (GB) distortion. (**b**) are the gradient images. (**c**) are the contrast-sensitive weighted gradient images.

**Figure 4:**
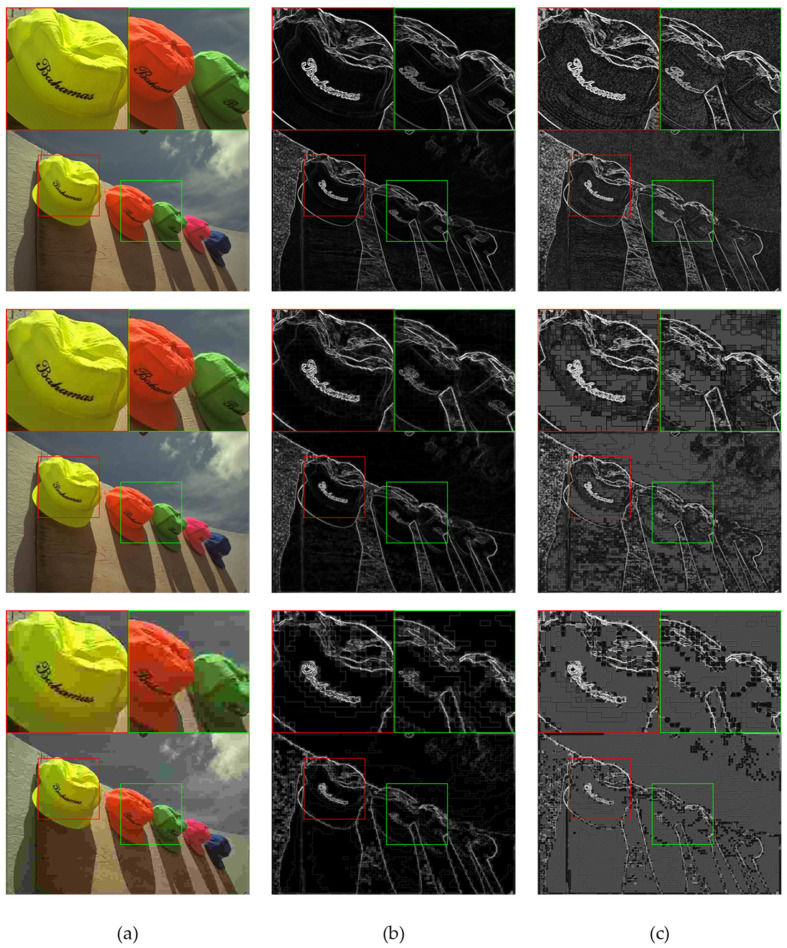
Examples of gradient images and the corresponding contrast-sensitive weighted gradient images for different JPEG distortion levels. (**a**) are JPEG distortion images at different levels, with DMOS scores of 12.56, 33.97, and 70.02 from top to bottom. A higher DMOS score indicates worse quality. (**b**) are the gradient images. (**c**) are the contrast-sensitive weighted gradient images.

Representative gradient images and the corresponding contrast-sensitivity-weighted gradient images are shown in Figure 3. Compared to the gradient images, it can be observed that the contrast-sensitivity-weighted gradient images better highlight the regions of interest to human eyes, such as the patterns around the eyes and the edges of the bird’s beak and body. This is because the contrast-sensitive weighted gradient images assign different weights to different regions of the images, which enables it to capture the image details that human eyes pay attention to. Additionally, contrast-sensitive weighted gradient images are also capable of capturing the structural information of distortions, such as block artifacts caused by JPEG compression and image noise caused by Gaussian distortion. These can significantly affect image quality. Figure 4 shows the gradient images and the corresponding contrast-sensitive weighted gradient images for different distortion levels of JPEG. It can be seen that, as the image distortion level increases from top to bottom, the block artifacts caused by JPEG compression become increasingly apparent, leading to declining image quality. For different distortion levels, contrast-sensitivity-weighted images can accurately capture the changes in distortion structures in the image, especially in highly sensitive regions of the HVS.

**Figure 5:**
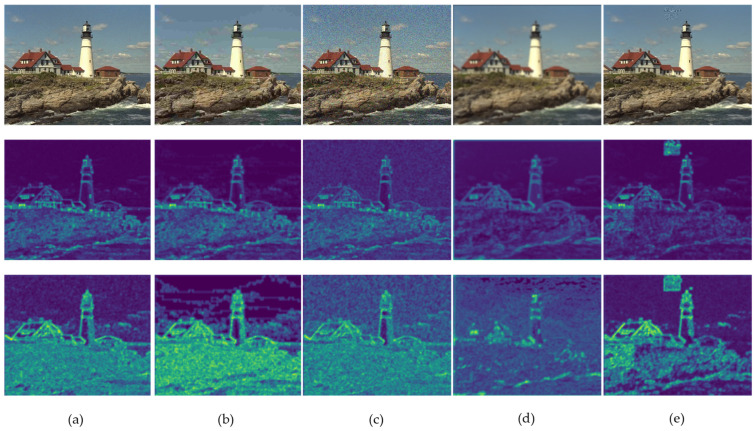
Examples of feature maps extracted by the “what” pathway and the “where” pathway on different distortion types, where the images are, from top to bottom, distorted images, feature maps extracted by the “what” pathway, and feature maps extracted by the “where” pathway, respectively. (**a**) is JP2K-compression distortion, (**b**) is JPEG-compression distortion, (**c**) is WN distortion, (**d**) is GB distortion, and (**e**) is FF distortion.

The feature maps extracted by the “what” pathway and the “where” pathway on different distortion types are shown in Figure 5. It can be seen intuitively that the feature maps extracted by the “what” pathway focus more on the content of the image, such as the lighthouse and buildings. The feature maps extracted by the “where pathway” not only focus on the shape of the main content but also accurately perceives the global shape of the image. This makes the “where” pathway able to accurately extract the distorted structural features in the image and enhance them. For example, the block effect is strengthened in the feature map extracted by JPEG distortion, the global noise is strengthened in the feature map extracted by WN distortion, and the blur-effect areas are more focused in the feature map extracted by GB distortion. Overall, the feature maps extracted by the “what” pathway focus more on the main content of the image, while the “where” pathway focuses more on global shape perception, rather than just the main features of the image. This is consistent with the function of the ventral and dorsal pathways and improves the performance of the model.

### 3.2. Proposed Multi-Scale Module

When the image quality is evaluated, the HVS not only focuses on the global content features of the image, which are the high-level features, but also pays attention to the local distortion features of the image, which are the low-level features [23]. This characteristic is particularly important for IQA tasks because it is often not uniformly distributed for the degree of distortion in images that have undergone authentic distortion. Using only global features may not enable the model to perceive the local distortion features of the image. Therefore, we propose a multi-scale module to extract distortion features of different scales in distorted images and effectively fuse the multi-scale features from the two streams. This enables the model to focus on both global and local features simultaneously, which is more in line with HVS perception.

**Figure 6:**
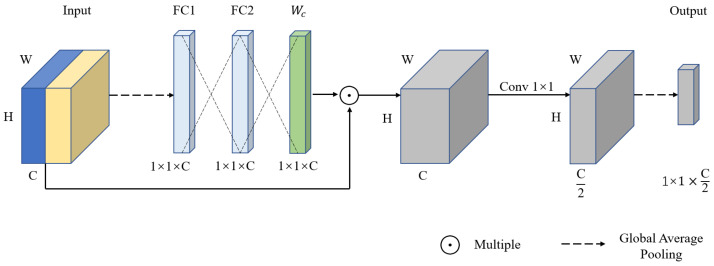
The structure of the proposed multi-scale module. Where W, H, and C represent the width, height, and channels of the feature map, respectively.

The multi-scale module, as shown in Figure 6, concatenates the features output by Conv2_10, Conv3_12, and Conv4_18 in the “what” pathway and the “where” pathway. A channel attention mechanism [52] is then used to reassign different channel importance to the concatenated feature map. Specifically, the concatenated feature map is, first, global-average-pooled to a one-dimensional vector. Then, a fully connected layer is used to generate a weight vector $W_{c}$ for each channel, so that each channel has a corresponding weight to better distinguish the importance of each channel. Finally, the weight vector is multiplied with the concatenated feature map to further fuse the feature maps from the “what” pathway and the “where” pathway, thereby enhancing the representational power and robustness of the features. A 1 × 1 convolution is used to reduce the number of channels in the fused feature map by half to reduce the computational cost, and global average pooling is applied to obtain a multi-scale feature vector. This process can be described as:

(6) $$\left\{\begin{array}{l} F_{i}=F_{c}⨂F_{p}\\ W_{c}=\sigma (W_{2}\delta (W_{1}GAP(F_{i})))\\ F_{i}^{\prime }=W_{c}F_{i}\\ F_{m}=GAP({Conv}_{1\times 1}(F_{i}^{\prime })) \end{array}\right.,$$

where $F_{c}$ and $F_{p}$ denote the feature maps from the “what” pathway and the “where” pathway, respectively. $W_{1}$ and $W_{2}$ are the parameters of two fully connected layers. σ(⋅) and δ(⋅) denote the sigmoid function and ReLU function. GAP(⋅) denotes global average pooling, and ${Conv}_{1\times 1}(\cdot )$ denotes 1×1 convolution operation.

### 3.3. Network Training

For data augmentation, we follow the training strategy in [11] and [53] by performing random horizontal flipping of the images in the training set and randomly sampling five 224 × 224-pixel image patches from each image to increase the number of training samples. The quality score of each image patch is the same as the quality score of the distorted image. Considering that the $L_{1}$ loss function is more robust to outliers, which is crucial in the task of image quality assessment, and the $L_{2}$ loss function is more sensitive to outliers, which can lead to poor fitting of exceptional samples during model training, the $L_{1}$ loss function is used to train the model:

(7) $$L_{1}=\frac{1}{N}\sum_{i=1}^{N}\left\|q_{i}-{\left.{\hat{q}}_{i}\right\|}_{l_{1}}\right.,$$

where $q_{i}$ represents the ground truth score of the image patch, ${\hat{q}}_{i}$ represents the predicted quality score of the image patch by the model, N denotes the number of image patches, and $l_{1}$ denotes the $l_{1}$-norm.

We use the Adam [54] optimizer for model parameter optimization with a weight decay rate of $5\times 10^{-4}$. The model is trained for 50 epochs with a batch size of 48, and the initial learning rate is set to $5\times 10^{-5}$, which is reduced by half every 10 epochs. During the testing process, we also randomly sample five 224 × 224 image patches from each testing image and calculate the average predicted quality score for five image patches as the quality score of the testing image. The proposed method is implemented by Pytorch and the experiments are conducted on an NVIDIA 3080Ti GPU.

## 4. Experiments

### 4.1. Image Quality Databases

To evaluate the performance of the proposed method, experiments are conducted on both synthetically distorted databases and authentically distorted databases, and the proposed approach is compared with the state-of-the-art methods. The synthetically distorted databases are LIVE [28], CSIQ [24], TID2013 [29], KADID-10k [30], and the Waterloo Exploration Database [44], with detailed information summarized in Table 2. The authentically distorted databases are LIVE Challenge (LIVEC) [31] and KonIQ-10k [55]. The LIVEC database contains 1162 images captured by different photographers using different equipment in natural environments, which include complex authentic distortion types. The KonIQ-10k dataset contains 10,073 images selected from the YFCC100M database [56], ensuring diversity in image content and quality, and an even distribution in brightness, color, contrast, and sharpness.

### 4.2. Experimental Protocols and Evaluation Metrics

To avoid content overlap between the training and testing images, we use 80% of the synthetically distorted databases based on the reference images for training and the remaining 20% for testing. For the authentically distorted databases, we directly use 80% of all images for training and 20% for testing. Each database is randomly split 10 times according to the aforementioned rule for experiments, and the average of 10 experimental results is taken as the final result.

We use the Spearman rank-order correlation coefficient (SROCC) and Pearson linear correlation coefficient (PLCC) to evaluate the performance of the IQA methods. These coefficients are used to evaluate the monotonicity and linear correlation between the predicted scores and the ground truth scores, respectively. Their range is [−1, 1], and the larger the absolute value is, the better the model’s performance is. In addition, on the Waterloo Exploration Database, the D-test metric is used to evaluate the model’s ability to distinguish between reference images and distorted images, the L-Test metric is used to evaluate the consistency between the predicted rank orders with different distortion levels but the same content and distortion type and their true rank orders, and the P-Test metric is used to evaluate the consistency of the IQA model in terms of the distortion score order between image pairs and their true order.

### 4.3. Performance on Individual Database

The experimental results on the individual databases are summarized in Table 3 and Table 4. The proposed method is compared with three traditional methods (PSNR, SSIM [57], and BRISQUE [5]) and seven deep-learning-based methods (IQA-CNN [8], BIECON [33], MEON [35], DIQaM-NR [9], HyperIQA [11], MMMNet [58], AIGQA [59], DB-CNN [12], and TS-CNN [13]) in terms of SROCC and PLCC results on six databases. Here, the DB-CNN and TS-CNN are similar to our proposed method, both with a dual-stream structure.

From Table 3 and Table 4, it can be observed that all methods exhibit good performance on the LIVE and CSIQ databases, which contain fewer distortion types. However, varying degrees of performance degradation are evident on the more complex distortion types of the TID2013 and KADID-10k databases, as well as the synthetically distorted databases of LIVEC and KonIQ-10k.

On the synthetically distorted databases of LIVE, TID2013, and KADID-10k, the proposed method achieves the top two SROCC and PLCC scores. On the authentically distorted databases of LIVEC and KonIQ-10k, the performance of the proposed method is among the top two methods, partly because the proposed method adopts a pre-trained ResNet-50 as the backbone to enable the model to learn the authentic distortions in the images more easily. Additionally, since the degree of distortion distribution in authentic distortion images is uneven, the proposed method introduces a multi-scale feature fusion module. This allows the model to focus on local details and better align with human visual perception.

Overall, based on the SROCC and PLCC results, the proposed method demonstrates excellent performance on six commonly used databases. Compared with other dual-pathway structures such as DB-CNN and TS-CNN, the proposed method maintains a leading position on most databases. In particular, compared with TS-CNN, the proposed method shows a significant performance difference on authentically distorted databases. This is mainly due to the incorporation of the dual-path characteristics of the HVS in the proposed approach, which can extract the content and location features of distorted images simultaneously. The contrast-sensitivity-weighted gradient image can explicitly extract the frequency information that is of interest to human vision. Additionally, the proposed multi-scale feature fusion module allows the model to focus on both global content and local details.

### 4.4. Performance on Individual Distortion Types

To compare the performance of the proposed method with the state-of-the-art methods on individual distortion types, experiments are conducted on three synthetically distorted databases, LIVE, CSIQ, and TID2013. All the distortion types are used for training on each database, and testing is performed on specific distortion types. The experimental results are summarized in Table 5, Table 6, and Table 7 for each database, respectively.

From Table 5, it can be observed that the proposed method achieves the best performance on four distortion types, JPEG, WN, GB, and FF, in the LIVE database. In particular, the proposed method outperforms other methods by a large margin on the FF distortion type. From Table 6, it can be seen that the proposed method achieves the best performance on four distortion types, JPEG, WN, PN, and CC, in the CSIQ database, and obtains the second- and third-best performance on the JP2K and GB distortion types, respectively, with only a small gap between it and the top methods. For the more complex distortion types of PN and CC, the proposed method still maintains a high SROCC.

It can be observed from Table 7 that the proposed method achieves top-two performance on 17 out of 24 distortion types, second only to HyperIQA’s 19 of out 24. Moreover, for complex distortion types such as NPN, BW, MS, and CC, most methods fail to achieve satisfactory results, while the proposed method still achieves relatively good performance. It can be seen that our method maintains stable and excellent performance on all distortion types in TID2013. Overall, the experimental results on the individual distortion types of the three datasets demonstrate that our method also performs well for specific distortion types.

### 4.5. Performance across Different Databases

Cross-database testing is a common method to test model generalizability. We conduct cross-database tests on four databases: LIVE, CSIQ, TID2013, and LIVEC. Specifically, we train the model on one database and test it on the others, such as training the model on the LIVE database and testing on the CSIQ, TID2013, and LIVEC databases, and so on. The SROCC results of the tests are summarized in Table 8.

From Table 8, it can be seen that the proposed method achieves the best performance in a total of eight cases, surpassing the DB-CNN’s three cases. When cross-database testing is conducted among the three synthetically distorted databases (LIVE, CSIQ, and TID2013), most methods achieve relatively good results. However, because synthetically distorted databases cannot fully simulate authentic distortion, many methods cannot achieve good performance on authentically distorted databases. Nevertheless, the proposed method still maintains good performance in such scenarios. Although it is trained on LIVE, CSIQ, and TID2013 and tested on LIVEC, it achieves the best performance. Similarly, when trained on LIVEC and tested on LIVE, CSIQ, and TID2013, our method also maintains good performance and achieves better results than other methods on TID2013. Although the performance on LIVE and CSIQ is slightly lower than DB-CNN, the proposed method still outperforms other methods and maintains a significant lead.

To further evaluate the generalization performance of the proposed method on large-scale databases, we train the model on the entire LIVE database and test it on the Waterloo Exploration Database, calculating the D-Test, P-Test, and L-Test metrics. The experimental results are presented in Table 9. It can be observed that the proposed method achieves top-two performance in both D-Test and L-Test metrics. It also demonstrates competitive performance in the P-Test metric, which further validates its superior generalization capability.

### 4.6. Ablation Experiments

To validate the effectiveness of the modules in the proposed method, ablation experiments are conducted on the LIVE, CSIQ, TID2013, and LIVEC databases. The “what” pathway, which only takes distorted images as the input, is used as the baseline model. Then, the “where” pathway is added, which takes gradient images as the input, followed by the contrast-sensitivity-weighted gradient image as the input in comparison, and finally the multi-scale module. The experimental results are summarized in Table 10. To further validate the significance of module contributions to model performance improvement, paired *t*-tests were conducted on various models in the ablation experiments. The experimental results are shown in Table 11, where 1, 0, and −1 represent the models in the corresponding row that are significantly better than, indistinguishable from, or worse than the models in the respective column. The confidence interval is set at 95%.

From Table 10 and Table 11, it can be observed that when there is only one pathway in the model, the performance is poor, especially when it only contains the “where” pathway. This is because the model can only extract high-frequency information from the gradient image and lacks detail information. When the model contains both the “what” pathway and the “where” pathway, the model can extract rich structural information from the gradient domain of the distorted image and significantly improve the model’s performance. This improves the performance by 0.011, 0.019, 0.017, and 0.009 on the three databases, respectively. When the contrast-sensitivity-weighted gradient image is used as the input for the “where” pathway, the improvement in model performance is even more significant, with increases of 0.015, 0.028, 0.028, and 0.019 on the three databases, respectively. This demonstrates that using the contrast-sensitivity-weighted gradient map as input can explicitly make the model focus more on the sensitive parts of the HVS, making the model highly consistent with the HVS perception.

Then, when the multi-scale module without a channel attention mechanism is added to the dual-pathway model, a slight improvement in model performance can be observed. However, this improvement is not significant, as only a simple concatenation of feature maps from the two pathways is performed in this case. This may result in redundant or irrelevant information being combined, limiting the model’s ability to effectively leverage the complementary strengths of the two pathways. Finally, adding the multi-scale module with a channel attention mechanism to the model shows that the performance of both the “where” pathway and the “what” pathway, which take the gradient image and contrast-sensitivity-weighted gradient map as inputs, are improved, with the largest improvement seen on the authentically distorted database LIVEC, with increases of 0.011 and 0.014, respectively. This is because, by incorporating a channel attention mechanism, the model gains the ability to selectively attend to informative channels from both pathways, effectively enhancing the fusion process. This allows the model to capture more fine-grained relationships between different channels, leading to improved performance.

## 5. Conclusions

In this paper, we propose a dual-pathway CNN model for BIQA based on the dual-pathway characteristic and contrast sensitivity of the HVS. Both pathways use pre-trained ResNet-50 as a feature extractor to enhance their feature-extraction capability. The model can be used to evaluate the image quality of both synthetic and authentic distortions. Considering the contrast sensitivity and edge sensitivity of the HVS, the method uses contrast-sensitivity-weighted gradient images as the input to the “where” pathway, enabling the model to explicitly focus on the highly salient parts of distorted images. Finally, a multi-scale module is proposed to focus on the global and local features of images simultaneously. Experimental results on individual databases and individual distortion types demonstrate that the proposed method’s performance is comparable to the state-of-the-art methods. Cross-database experiments and experiments on the Waterloo Exploration Database also demonstrate that the proposed method has a strong generalization performance.

Although the proposed method has achieved good performance on most commonly used databases, there is still room for further improvement in some aspects. For example, better feature fusion methods such as bilinear pooling or other fusion methods could be considered when global features from both pathways and global and local features are fused. In addition, how to combine more HVS characteristics highly related to image quality assessment tasks (e.g., the bandpass characteristic [63,64] and the masking effect [65]) with deep learning methods is also a future research direction.

## Figures and Tables

**Table 1:** The architecture of ResNet-50.

Conv1	Conv2_10	Conv3_12	Conv4_18	Conv5_9
7 × 7, 64, s2	3 × 3 max pool, s2	$\left[\begin{array}{c} 1\times 1,128\\ 3\times 3,128\\ 1\times 1,512 \end{array}\right]\times 4$	$\left[\begin{array}{c} 1\times 1,256\\ 3\times 3,256\\ 1\times 1,1024 \end{array}\right]\times 6$	$\left[\begin{array}{c} 1\times 1,512\\ 3\times 3,512\\ 1\times 1,2048 \end{array}\right]\times 3$
	$\left[\begin{array}{c} 1\times 1,64\\ 3\times 3,64\\ 1\times 1,256 \end{array}\right]\times 3$			

**Table 2:** Details of the synthetically distorted databases.

Database	Ref. Imgs	Dist. Imgs	Dist. Types	Score’s Type
LIVE [28]	29	779	5	DMOS
CSIQ [24]	30	866	6	DMOS
TID2013 [29]	25	3000	24	MOS
KADID-10k [30]	81	10,125	25	DMOS
Waterloo [44]	4744	94,880	4	/

**Table 3:** The SROCC results on six databases. The top two results are shown in bold font.

SROCC	LIVE	CSIQ	TID2013	KADID-10k	LIVEC	KonIQ-10k
PSNR	0.866	0.806	0.636	0.674	-	-
SSIM [57]	0.913	0.876	0.637	0.783	-	-
BRISQUE [5]	0.940	0.746	0.604	0.519	0.607	0.673
IQA-CNN [8]	0.956	0.876	0.701	0.651	0.516	0.655
BIECON [33]	0.961	0.825	0.717	0.685	0.595	0.618
MEON [35]	0.943	0.839	0.828	0.813	0.693	0.754
DIQaM-NR [9]	0.960	0.901	0.835	0.840	0.606	0.722
HyperIQA [11]	0.962	0.923	0.840	0.852	**0.859**	**0.906**
MMMNet [58]	**0.970**	0.924	0.832	0.841	0.852	0.867
AIGQA [59]	0.960	0.927	**0.871**	**0.864**	0.751	0.766
DB-CNN [12]	0.968	**0.946**	0.816	0.801	0.851	0.875
TS-CNN [13]	0.969	0.892	0.779	0.745	0.655	0.722
DPCS	**0.971**	**0.929**	**0.866**	**0.882**	**0.856**	**0.909**

**Table 4:** The PLCC results on six databases. The top two results are shown in bold font.

PLCC	LIVE	CSIQ	TID2013	KADID-10k	LIVEC	KonIQ-10k
PSNR	0.856	0.800	0.706	0.681	-	-
SSIM [57]	0.931	0.861	0.691	0.780	-	-
BRISQUE [5]	0.942	0.829	0.694	0.554	0.585	0.692
IQA-CNN [8]	0.953	0.905	0.752	0.607	0.536	0.671
BIECON [33]	0.962	0.838	0.762	0.691	0.613	0.651
MEON [35]	0.954	0.850	0.811	0.822	0.688	0.760
DIQaM-NR [9]	0.972	0.908	0.855	0.843	0.601	0.736
HyperIQA [11]	0.966	0.942	0.858	0.845	**0.882**	**0.917**
MMMNet [58]	0.970	0.937	0.853	0.840	0.846	0.871
AIGQA [59]	0.957	**0.952**	**0.893**	**0.863**	0.761	0.773
DB-CNN [12]	0.971	**0.959**	0.865	0.806	0.869	0.884
TS-CNN [13]	**0.978**	0.905	0.784	0.744	0.667	0.729
DPCS	**0.973**	0.935	**0.880**	**0.884**	**0.873**	**0.914**

**Table 5:** The SROCC results of the individual distortion type on the LIVE database. The top two results are shown in bold font.

SROCC	JP2K	JPEG	WN	GB	FF
PSNR	0.870	0.885	0.942	0.763	0.874
SSIM [57]	0.939	0.946	0.964	0.907	**0.941**
BRISQUE [5]	0.910	0.919	0.955	0.941	0.874
IQA-CNN [8]	0.936	0.965	0.974	0.952	0.906
BIECON [33]	0.952	**0.974**	0.980	0.956	0.923
MEON [35]	0.953	0.964	0.981	0.958	0.904
DIQaM-NR [9]	0.914	0.951	0.972	0.944	0.926
HyperIQA [11]	0.949	0.961	0.982	0.926	0.934
DB-CNN [12]	0.955	0.972	0.980	0.935	0.930
TS-CNN [13]	**0.966**	0.950	0.979	**0.963**	0.911
MMMNet [58]	**0.968**	**0.974**	**0.985**	0.935	0.936
DPCS	0.963	**0.978**	**0.987**	**0.966**	**0.957**

**Table 6:** The SROCC results of the individual distortion type on the CSIQ database. The top two results are shown in bold font.

SROCC	JP2K	JPEG	WN	GB	PN	CC
PSNR	0.926	0.888	0.936	0.829	0.874	0.852
SSIM [57]	0.921	0.922	0.925	0.914	0.941	0.740
BRISQUE [5]	0.840	0.806	0.723	0.820	0.378	0.804
IQA-CNN [8]	0.930	0.915	0.919	0.918	0.900	0.786
BIECON [33]	0.954	0.942	0.902	0.946	0.884	0.523
MEON [35]	0.934	0.922	0.944	0.901	0.867	0.847
DIQaM-NR [9]	0.896	**0.946**	0.947	0.908	0.895	0.807
HyperIQA [11]	**0.960**	0.934	0.927	0.915	0.931	0.874
DB-CNN [12]	**0.953**	0.940	**0.948**	**0.947**	0.940	0.870
TS-CNN [13]	0.914	0.907	0.938	0.895	0.882	0.866
MMMNet [58]	0.932	0.912	0.879	0.894	**0.941**	**0.942**
DPCS	0.936	**0.947**	**0.954**	**0.930**	**0.944**	**0.912**

**Table 7:** The SROCC results of the individual distortion types on the TID2013 database. The top two results are shown in bold font.

SROCC	BRISQUE [5]	IQA-CNN [8]	MEON [35]	DIQA [10]	HyperIQA [11]	DB-CNN [12]	TS-CNN [13]	DPCS
AGN	0.711	0.784	0.813	**0.916**	**0.942**	0.790	0.816	0.890
ANC	0.432	0.758	0.722	0.755	**0.916**	0.700	0.704	**0.794**
SCN	0.746	0.762	0.926	0.878	**0.947**	0.826	0.809	**0.960**
MN	0.252	0.776	0.728	0.734	**0.801**	0.646	0.475	**0.848**
HFN	0.842	0.816	0.911	**0.939**	**0.955**	0.879	0.833	0.906
IN	0.765	0.807	**0.901**	0.844	0.855	0.708	0.819	**0.899**
QN	0.662	0.616	**0.888**	0.858	0.726	0.825	0.801	**0.873**
GB	0.871	**0.921**	0.887	0.920	**0.969**	0.859	0.786	0.858
DEN	0.612	0.872	0.797	0.788	**0.941**	0.865	0.733	**0.871**
JPEG	0.764	0.874	0.850	0.892	**0.898**	0.894	0.847	**0.896**
JP2K	0.745	0.910	0.891	0.812	**0.947**	**0.916**	0.851	0.909
JGTE	0.301	0.686	0.746	**0.862**	**0.934**	0.772	0.699	0.843
J2TE	0.748	0.678	0.716	0.813	**0.892**	0.773	0.766	**0.894**
NPN	0.269	0.286	0.116	0.160	**0.808**	0.270	0.211	**0.600**
BW	0.207	0.219	**0.500**	0.408	0.361	0.444	0.313	**0.639**
MS	0.219	**0.565**	0.177	0.300	0.374	−0.009	0.107	**0.545**
CC	−0.001	0.182	0.252	0.447	**0.753**	0.548	0.315	**0.819**
CCS	0.003	0.081	0.684	0.151	**0.857**	0.631	0.324	**0.725**
MGN	0.717	0.644	0.849	**0.904**	0.899	0.711	0.744	**0.910**
CN	0.196	0.534	0.406	0.656	**0.960**	0.752	0.638	**0.849**
LCNI	0.609	0.810	0.772	0.830	**0.897**	0.860	0.742	**0.918**
ICQD	0.831	0.272	0.857	**0.937**	**0.901**	0.833	0.759	0.872
CHA	0.615	**0.892**	0.779	0.757	**0.870**	0.732	0.714	0.823
SSR	0.807	0.910	0.855	0.909	**0.910**	0.902	0.826	**0.933**
Mean	0.538	0.652	0.709	0.728	**0.846**	0.714	0.651	**0.836**
Count	0	3	3	5	**19**	1	0	**17**

**Table 8:** The SROCC results of cross-database tests. The top result is shown in bold font.

Training	LIVE			TID2013		
Testing	CSIQ	TID2013	LIVEC	LIVE	CSIQ	LIVEC
DIIVINE [6]	0.582	0.373	0.300	0.714	0.585	0.230
BRISQUE [5]	0.562	0.358	0.326	0.758	0.570	0.209
CORNIA [60]	0.620	0.382	0.431	0.829	0.662	0.267
HOSA [61]	0.598	0.470	0.455	0.844	0.609	0.253
IQA-CNN [8]	0.616	0.407	0.103	0.530	0.600	0.102
DIQaM-NR [9]	0.623	0.425	0.206	0.812	0.698	0.112
DB-CNN [12]	0.758	0.524	0.567	0.891	**0.807**	0.457
TS-CNN [13]	0.621	0.431	0.273	0.576	0.609	0.114
MMMNet [58]	**0.793**	0.546	0.502	0.853	0.702	0.348
DPCS	0.743	**0.614**	**0.587**	**0.897**	0.739	**0.462**
**Training**	**CSIQ**			**LIVEC**		
**Testing**	**LIVE**	**TID2013**	**LIVEC**	**LIVE**	**CSIQ**	**TID2013**
DIIVINE [6]	0.815	0.419	0.366	0.362	0.417	0.337
BRISQUE [5]	0.790	0.590	0.106	0.346	0.245	0.258
CORNIA [60]	0.843	0.331	0.393	0.578	0.456	0.403
HOSA [61]	0.770	0.341	0.309	0.537	0.336	0.399
IQA-CNN [8]	0.713	0.315	0.103	0.213	0.195	0.132
DIQaM-NR [9]	0.817	0.516	0.114	0.319	0.313	0.215
DB-CNN [12]	0.877	0.540	0.452	**0.746**	**0.697**	0.424
TS-CNN [13]	0.836	0.477	0.158	0.283	0.249	0.225
MMMNet [58]	0.890	0.522	0.406	0.528	0.518	0.398
DPCS	**0.893**	**0.584**	**0.491**	0.638	0.686	**0.426**

**Table 9:** Results of D-Tests, L-Tests, and P-Tests. The top two results are shown in bold font.

Method	D-Test	L-Test	P-Test
BRISQUE [5]	0.920	0.977	0.993
IQA-CNN [8]	0.929	0.930	0.997
dipIQ [62]	0.935	**0.985**	**0.999**
DIQaM-NR [9]	0.907	0.947	0.963
MEON [35]	0.938	0.967	0.998
HyperIQA [11]	0.901	0.975	0.997
DB-CNN [12]	**0.962**	0.961	**0.999**
TS-CNN [13]	0.930	**0.979**	0.995
DPCS	**0.941**	0.976	**0.999**

**Table 10:** The SROCC results of the ablation experiments. The top result is shown in bold font.

Baseline	√			√	√	√	√	√	√
Gradient Image		√	√	√	√	√	√	√	√
Contrast Sensitivity			√		√			√	√
Multi-scale Module							√		√
Multi-scale Module w/o CA						√		√	
LIVE	0.951	0.938	0.944	0.962	0.966	0.963	0.968	0.967	**0.971**
CSIQ	0.894	0.864	0.875	0.913	0.922	0.916	0.921	0.924	**0.929**
TID2013	0.832	0.806	0.814	0.849	0.860	0.851	0.854	0.862	**0.866**
LIVEC	0.823	0.768	0.773	0.832	0.842	0.837	0.843	0.848	**0.856**

**Table 11:** The *t*-test results of the different models in ablation experiments. M1 to M9 correspond to the models in each column of Table 10, respectively.

Model	M1	M2	M3	M4	M5	M6	M7	M8	M9
M1	0	1	1	−1	−1	−1	−1	−1	−1
M2	−1	0	−1	−1	−1	−1	−1	−1	−1
M3	−1	1	0	−1	−1	−1	−1	−1	−1
M4	1	1	1	0	−1	−1	−1	−1	−1
M5	1	1	1	1	0	1	0	−1	−1
M6	1	1	1	1	−1	0	−1	−1	−1
M7	1	1	1	1	0	1	0	0	−1
M8	1	1	1	1	1	1	0	0	−1
M9	1	1	1	1	1	1	1	1	0

## Data Availability

Not applicable.
